# Preclinical translational platform of neuroinflammatory disease biology relevant to neurodegenerative disease

**DOI:** 10.1186/s12974-024-03029-3

**Published:** 2024-01-31

**Authors:** Kelley C. Larson, Lauren H. Martens, Michael Marconi, Christopher Dejesus, Suzanne Bruhn, Thomas A. Miller, Barbara Tate, Jonathan M. Levenson

**Affiliations:** 1 Present Address: Vigil Neuroscience, Watertown, USA; 2 Present Address: Neumora Therapeutics, Watertown, USA; 3 Present Address: Department of Molecular Pathology, Massachusetts General Hospital, Boston, USA; 4 Present Address: Atalanta Therapeutics, Boston, USA; 5 Present Address: Charcot-Marie-Tooth Association, Glenolden, USA; 6 Present Address: Walden Biosciences, Cambridge, USA; 7 Present Address: FARA, Homestead, USA; 8 Present Address: FireCyte Therapeutics, Beverly, USA; 9 Tiaki Therapeutics, Inc., c/o Dementia Discovery Fund, 201 Washington Street, 39th Floor, Boston, MA 02108 USA

**Keywords:** Microglia, Neurodegeneration, Neuroinflammation, Organotypic

## Abstract

Neuroinflammation is a key driver of neurodegenerative disease, however the tools available to model this disease biology at the systems level are lacking. We describe a translational drug discovery platform based on organotypic culture of murine cortical brain slices that recapitulate disease-relevant neuroinflammatory biology. After an acute injury response, the brain slices assume a chronic neuroinflammatory state marked by transcriptomic profiles indicative of activation of microglia and astrocytes and loss of neuronal function. Microglia are necessary for manifestation of this neuroinflammation, as depletion of microglia prior to isolation of the brain slices prevents both activation of astrocytes and robust loss of synaptic function genes. The transcriptomic pattern of neuroinflammation in the mouse platform is present in published datasets derived from patients with amyotrophic lateral sclerosis, Huntington’s disease, and frontotemporal dementia. Pharmacological utility of the platform was validated by demonstrating reversal of microglial activation and the overall transcriptomic signature with transforming growth factor-β. Additional anti-inflammatory targets were screened and inhibitors of glucocorticoid receptors, COX-2, dihydrofolate reductase, and NLRP3 inflammasome all failed to reverse the neuroinflammatory signature. Bioinformatics analysis of the neuroinflammatory signature identified protein tyrosine phosphatase non-receptor type 11 (PTPN11/SHP2) as a potential target. Three structurally distinct inhibitors of PTPN11 (RMC-4550, TN0155, IACS-13909) reversed the neuroinflammatory disease signature. Collectively, these results highlight the utility of this novel neuroinflammatory platform for facilitating identification and validation of targets for neuroinflammatory neurodegenerative disease drug discovery.

**Supplementary Information:**

The online version contains supplementary material available at 10.1186/s12974-024-03029-3.

## Introduction

Neuroinflammation is increasingly recognized as a vital component of the overall biology underlying neurodegenerative disease [1–3]. Retrospective analyses of post-mortem brain tissue has revealed the presence of sizeable subpopulations of patients that exhibit neuroinflammation in the context of Alzheimer’s disease [4], Amyotrophic lateral sclerosis [5], Parkinson’s disease [6], frontotemporal dementia [7], and vascular dementia [8]. Several clinical studies have shown significant correlations between PET-imaging ligands sensitive to activated microglia and astrocytes, and changes in disease-relevant neurodegenerative pathologies, suggesting a causal link between neuroinflammation and progression of disease [9–14]. Despite the mounting evidence, there are few translational tools available that accurately model human disease-relevant neuroinflammatory biology in a preclinical setting to facilitate target validation and drug discovery activities.

Microglia are the resident immune cells of the CNS and subserve numerous supportive functions, contributing to diverse processes such as neurodevelopment, synaptic maintenance and remodeling, synaptic plasticity, and promotion of myelination [15–19]. Their highly plastic and communicative nature permits the breadth of functionality exhibited by microglia, but also likely explains why microglia cultured in vitro rapidly lose their in vivo phenotype [20]. Efforts to create in vivo-relevant model systems for neuroinflammation have often relied on co-culture systems that contain neurons and/or astrocytes [21, 22]. While these approaches begin to model the systems biology that contributes to neuroinflammation, no culture system has been confirmed to recapitulate the transcriptomic signatures of disease-relevant microglial activation [23–25]. Additionally, age-related changes in distinct microglial phenotypes within the population of the murine CNS [26] make assay reproducibility, as well as comparisons between different labs and protocols, extremely challenging. This highlights the critical need for more robust translational tools that model microglia in a state that is both in vivo-relevant and aligned with human disease.

Alternative approaches to creation of translational neuroinflammatory platforms have focused on in vivo and ex vivo platforms. Several independent laboratories have demonstrated successful creation of chimeric mice where murine microglia are replaced with human iPSC-derived microglia cells [27–29]. Chimeric microglia mouse models have been shown to maintain human microglia in an in vivo-relevant state in which microglia develop human disease-relevant changes as assessed through transcriptomics. While promising, these mouse models require multiple genetic modifications to support the transplanted human iPSC-derived microglia, with more genetic modifications required to model neurodegenerative disease-relevant pathology. In addition, these models require depletion of endogenous murine microglia, surgical transplantation of the human microglial cells, and subsequent aging to ensure full repopulation by the human microglia and development of pathologies. Lastly, the implanted human microglia tend to have short lifespans, unlike native microglial populations. This makes implementation of these translational models in a drug discovery environment impractical. More recent approaches to translational models for microglial biology revealed that organotypic culture of brain slices from early postnatal mouse brain (p1–p4) represents a viable system for recapitulating adult, human-like microglia [30]. While promising, this slice culture system requires exogenous pharmacological agents to induce neuroinflammation, which could bias the types of neuroinflammatory biology this system models. The approach by Delbridge ARD*, *et al*.* [30] also highlights a potential shortcoming of approaches leveraging early postnatal rodents such as Pischiutta F*, *et al*.* [31] (p1–p3 mice) and Kamikubo Y*, *et al*.* [32] (p6–p8 rats). Young microglia tend to be in a different functional state relative to adult microglia with more regenerative capacity, which could skew the spectrum of biology modeled in these systems [33, 34]. Recent progress in technologies that combine human IPSC-derived microglia, neurons, and astrocytes cultured on synthetic matrices such as the “brain-chip” [35] could begin to recapitulate the systems biology involved in induction and maintenance of chronic neuroinflammation. However, none of these organoid-based systems has been able to fully recapitulate adult, CNS-relevant neuroinflammatory biology to date [36].

This study investigates the characteristics of murine organotypic brain slice cultures derived from young adult mice. The hypothesis behind this approach was that these cultures would lack some of the neuronal regenerative capacities of organotypic brain slice cultures derived from early postnatal mice [37–41]. We show that our platform, microglia in organotypic brain slice cultures derived from adult mice initially exhibit a robust injury response that resolves into a chronic neuroinflammatory state, which exemplifies the previously described disease-associated microglial functional state [23]. Bulk transcriptomic profiling reveals a profound loss of neuronal gene expression upon entry into the chronic neuroinflammatory state. This chronic neuroinflammatory state required the presence of microglia, as the activation of astrocytes did not occur in the absence of microglia. In addition, analysis of conditioned media revealed a pattern of cytokine secretion that reflects the transcriptomic changes that were observed. Importantly, transcriptomic changes in slice cultures during the chronic neuroinflammatory phase aligned with the publicly available human transcriptomes from subpopulations of patients with neurodegenerative disorders that exhibit neuroinflammation. Collectively, these results demonstrate that brain slices from adult mice under optimized organotypic culture conditions recapitulate human disease-relevant neuroinflammation without pharmacological or genetic intervention, making them an ideal platform for the identification and interrogation of biological processes involved in neurodegenerative neuroinflammatory processes.

## Methods

### Animals

All studies used 21-day-old C57BL/6 MPF mice (Taconic Biosciences). Equal numbers of male and female mice were used in these studies. Mice assigned for organotypic slice cultures were used within 3 h of arriving at the vivarium. Mice used in microglia depletion studies were housed in 12:12 LD cycles, with ad libitum access to chow and water. In some cages, chow was formulated with PLX3397 (PLX3397 acquired from Tocris [#7590]) to achieve approximately 40 mg/kg/day dosing as previously described (290 mg PLX3397/kg chow, formulated by Research Diets) [42]. After 10 days of exposure to PLX3397 or normal chow, mice were killed for further experimentation and analyses. No change in animal weights was observed, consistent with other reports of systemic dosing with PLX3397 [43].

### Organotypic brain slice culture

Mice were euthanized by CO_2_ inhalation and immediately decapitated. Brains were removed and transferred to cold “Dissection Media”, containing Hibernate A (Gibco #A1247501), 20% heat inactivated horse serum (Sigma, #H1138), antimycotic/antibiotic (Anti/Anti, Gibco, #15,240–062), and glucose solution (Gibco, #A2494001) to 10 mM final Brains were then transferred to a cold block, cerebellum removed, and hemispheres were separated with a sterile razor blade longitudinally, before being placed on a sterile disk and attached with Vetbond in a Tissue Chopper (McIllwain). 300-µm slices were generated, and the entire brain was removed and returned to a fresh dish of cold dissection media to recover for 5 min. Individual slices (2 per well) were transferred to a 6-well plate containing 30 mm cell culture inserts (PICM03050) with 1.2 mL of prewarmed culture media containing Neurobasal media (Gibco, #10,888–022), 20% heat inactivated horse serum, Anti-Anti, and 10 mM glucose. All cultures were prepared from equal amounts of tissue from male and female mice. Plates were incubated overnight in a humidified incubator, 37 °C, 5% CO_2_. On days 1 and 3, 50% of the media was removed and replaced with freshly made culture media. On day 5, media was replaced completely with serum-free media containing neurobasal media, 2% B27 (containing Vitamin A, Gibco, #17,504-044), anti-anti, and 10 mM glucose. Every 2–3 days therein, 50% of the media was removed and replaced with freshly made serum-free medium.

TGF-β2 treatments. Cultures were treated with vehicle (PBS) or recombinant murine TGF-β2 (250 ng/mL; Bio-Techne #: 7346-B2-005/CF) beginning on day in vitro (DIV)1. Bulk tissue was harvested on DIV6 and prepared for RNA-seq as detailed below. Fresh TGF-β2 was included in all scheduled media changes. Data are the average of 5 biological replicates.

Small molecule treatments. All treatments were initiated on DIV5 coincident with the scheduled media change. Organotypic slice cultures were exposed to vehicle (DMSO) or one of the following compounds: dexamethasone (5 µM, Tocris #: 1126/100), rofecoxib (1 µM, Selleckchem #: S3043), methotrexate (0.2 µM, Tocris #: 1230/100), CRID3 (20 µM, Tocris #: 5479/10), RMC-4550 (0.05 µM–5 µM, Selleckchem #: S8718), TNO155 (0.1 µM–1 µM, Selleckchem #: S8987), IACS-13909 (10 µM, Selleckchem #: S9703). Doses are 10x–100 × above published EC_50_ or IC_50_ derived from either biochemical or cell-based assays. Data reported are derived from 3 biological replicates. Each replicate consisted of tissue derived from four different mice (2 male, 2 female). Compounds were refreshed on DIV7 during the scheduled media change. All tissue were harvested on DIV8.

### Isolation of microglia

Microglia were isolated from six organotypic brain slices, pooled from 3 individual wells or from freshly isolated mouse brain tissue using the Miltenyi OCTOMacs system. Tissue was added to C-tubes containing buffers and enzymes according to Miltenyi specifications. After homogenization, the cell suspension was passed through a 30-micron cell strainer into prechilled, sterile tubes. Myelin was removed using myelin removal beads (Miltenyi) and LS columns on a MACS separator. All wash buffers were prechilled and used according to manufacturer recommendations. Microglia were isolated using Miltenyi CD11b + beads and MS columns. Microglia were eluted from the column, spun, and resuspended in appropriate buffer for subsequent experiments.

### Purification of mRNA

#### Isolated microglia

mRNA was isolated from purified microglia using TRIZol reagent. Briefly, TRIZol and chloroform were both added to cells, incubated, and spun at 4 °C for 15 min at 12,000 ×*g*. The aqueous layer was transferred to RNAse-free Eppendorf tubes containing ethanol. This mixture was then added to Qiagen RNeasy RNA columns (micro column). RNA was extracted and purified according to manufacturer instructions.

#### Bulk tissue

mRNA was isolated from six organotypic brain slices, pooled from 3 individual wells or mouse brain tissue using the Maxwell RSC 16 instrument and mammalian RNA isolation kits. Tissue was homogenized using disposable, sterile pestles and transferred to kit cartridges. Cartridges were loaded into the Maxwell automated system and run according to manufacturer’s instructions. Purified mRNA was eluted into RNAase free water.

#### mRNA QC

mRNA concentration was quantified using Nanodrop, and quality was assessed using the Agilent Bioanalyzer. Only samples with a RIN of ≥ 7 were used in subsequent transcriptomic analyses.

#### RNA-sequencing

Briefly, exome-enriched cDNA libraries were constructed using TruSeq RNA Exome (Illumina, San Diego, CA) according to the manufacturer’s recommendations. Libraries were loaded on a NextSeq 500 sequencing system (Illumina, San Diego, CA) with a high output v3 150 cycle reagent kit, with a mean of 25 million reads per sample. Base call files from each sequencing run were converted to fastq format using bcl2fastq conversion software (Illumina, San Diego, CA) and aligned to the Ensembl GRCh37 Homo sapiens reference using STAR (Spliced Transcripts Alignment to a Reference, v.020201). Transcript assembly and expression analysis were performed on each sample with cufflinks v. 2.2.1, resulting in fragments per kilobase million (FPKM) values for each transcript in the genes of interest, which were summed into one FPKM value for each gene [44, 45]. Data were Log_2_ transformed and normalized using Limma-Voom. Differential expression was calculated as Experiment–Control, and significance was determined using an adaptive linear step-up procedure [46] with a false discovery rate of 1%.

### Digital quantification of transcriptomic changes via NanoString

100 ng RNA was hybridized to Nanostring’s Mouse Neuropathology probe set, with a custom gene panel added, for 18 h overnight at 60 °C. Samples were then loaded into sprint cartridges, washed, and scanning using the NanoString Sprint according to manufacturer’s protocols. Counts were normalized against the geometric mean of 10 housekeeping genes, and Log_2_ fold changes determined against untreated controls.

### Bioinformatics

Principal component analyses (PCA) were calculated using normalized, Log2-transformed counts (Orange v3, [47]). Driver genes of the principal components were identified as genes that had a differential calculated from the waterfall plot of Eigenvectors that was greater than the 5th percentile of all differentials.

Gene set enrichment and transcription factor binding motif enrichment analyses were performed using g:Profiler [48]. Venn Diagrams were created using Orange (v3). Hierarchical clustering was performed using Manhattan distances and Ward linkage (Orange v3).

Bulk tissue transcriptomic data from patients with neurodegenerative disease were obtained from publicly available databases. ALS data were obtained from Tam OH*, *et al*.* [5], FTD data were obtained from GSE13162, and HD data were obtained from GSE3790. Transcriptomes were aligned by gene orthology and quantified using Pearson correlation.

In some heat maps, data are scaled to facilitate visualization. Scaling was done by multiplying Log_2_[Fold Change] by a factor that yields the sum of squares for the entire dataset equivalent to the number of genes detected.

### Secretomic analyses

Conditioned media was collected from cultures and flash frozen. Samples were diluted 1:2 in assay buffer and analyzed using the FirePlex-96 Discovery Cytokines (Mouse) Immunoassay (abcam, #ab243554, Waltham, MA) according to the manufacturer’s instructions. Patterns of differential secretion were determined by transforming longitudinal ng/mL measurements to Z-scores. Z-scores were further stratified and visualized using hierarchical clustering analysis (Manhattan distance, Ward linkage, Orange v3).

## Results

### Microglia from brain slice cultures assume a disease-like transcriptome

**Fig. 1:**
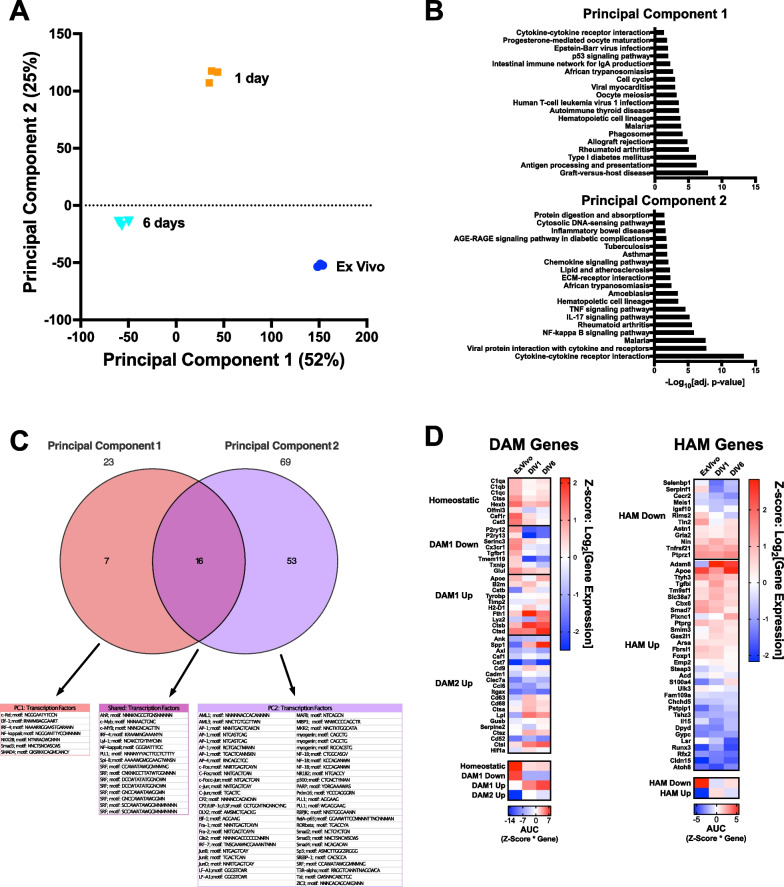
Transcriptomic changes in microglia indicate entry into a chronic neuroinflammatory state. RNA-seq was performed on microglia either freshly purified from mouse brain or isolated from organotypic brain slice cultures in three independent experiments after 1 or 6 days in organotypic culture. **A** Principal component analysis indicated good clustering of individual microglial preparations and robust separation of each time point. **B** Gene set enrichment analysis of driver genes for each principal component was performed to identify salient biology described by each axis. ‑Log_10_[adj p-val] for enriched Kegg pathways is shown. **C** Transcription factor enrichment of the driver genes was assessed for each principal component. Note the high representation of transcription factors associated with inflammation in Principal Component 1 and immediate early genes and microglial development in Principal Component 2. **D** The canonical murine disease-associated microglial gene panel [23] and human AD microglia gene panel were used to assess the functional state of microglia with time in organotypic culture. Shown are two heat maps of Z-scores derived from Log_2_[Fold Changes] (scale to the right of the heat map). Below each main heatmap are summary heatmaps indicating the area under the curve for each gene category. Within 1 day of culture, microglia adopted a strong DAM1 (Trem2-independent) state and shifted toward the HAM state. This pattern persisted into DIV6

Transcriptomes from microglia that were freshly isolated from mouse brain or organotypic brain slice cultures after 1 or 6 days in culture, were analyzed via RNA-seq. Dimensionality reduction via principal component analysis permitted identification of biological processes driving the longitudinal differences (Fig. 1A). Gene-set enrichment analysis of driver genes identified by principal component analysis indicated that after 1 day in culture, microglia from organotypic brain slice cultures exhibited a robust acute inflammatory response (Fig. 1A, B). This same analysis after 6 days in culture indicated that microglia isolated from brain slice cultures exhibited biological perturbations consistent with a chronic neuroinflammatory state (Fig. 1B). Specifically, functional pathways that describe phagocytic and chronic inflammatory processes were most significantly perturbed (Fig. 1B). Analysis of regulatory sites over-represented in the promoters of driver genes for each principal component indicated that principal component 1 was associated with transcription factors engaged during inflammation including Nf-κB, IRF-4, and SMAD (Fig. 1C). Transcription factor sites associated with principal component 2 are involved in immediate early response transcriptional programs and microglial development and identity (Fig. 1C) including AP-1, c-Jun, and c-Fos. To further characterize the change in functional state of microglia, the murine disease-associated microglia (DAM) gene panel initially described by Keren-Shaul H, et al*.* [23] and the human Alzheimer’s disease microglia (HAM) gene panel initially described by Srinivasan K, et al*.* [24] were used. Microglia isolated directly from naïve mouse brain exhibited a transcriptional fingerprint indicative of the homeostatic state and non-HAM state (Fig. 1D). Microglia isolated from brain slices after 1 or 6 days in culture exhibited a pattern of gene expression suggestive of a shift away from homeostasis and non-HAM, and a shift toward the murine DAM1 (Trem2-independent) disease state and the HAM state (Fig. 1D).

### Organotypic brain slice culture enters a stable, inflammatory state

**Fig. 2:**
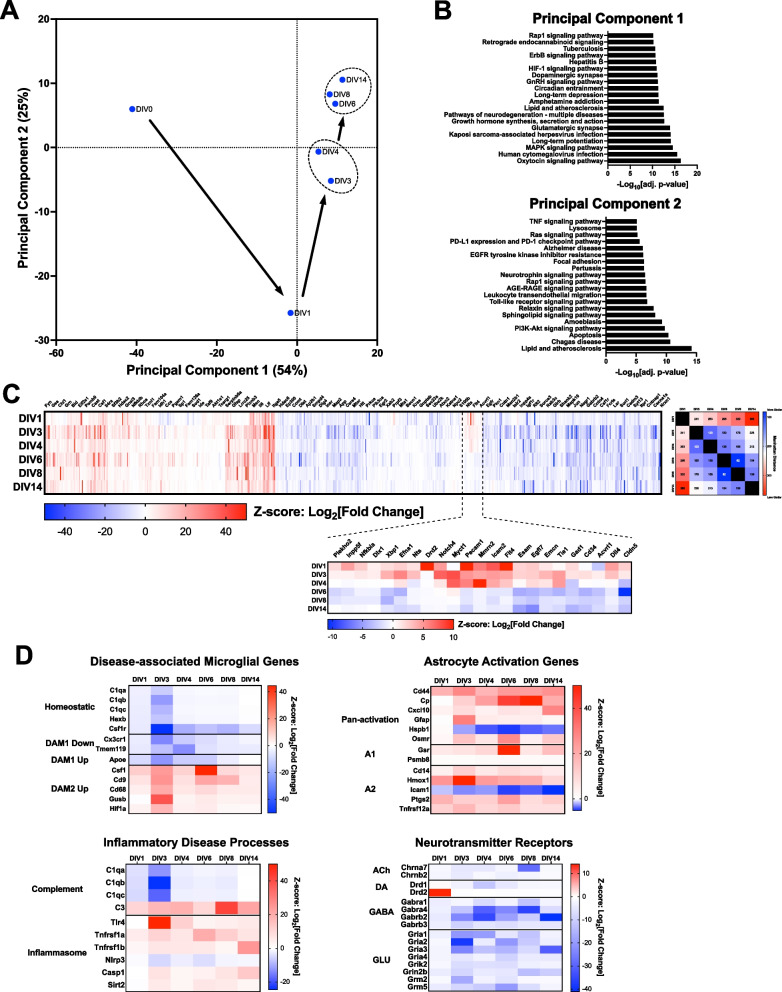
Transcriptomic changes in bulk tissue indicate ongoing microgliosis and astrocytosis associated with loss of synaptic function genes. Transcriptomic analysis using NanoString (Neuropathology panel) was performed on bulk cortical tissue from three independent experiments either freshly harvested from mouse brain or collected from brain slices after 1–14 days in organotypic culture. **A** Principal component analysis indicated a robust change in the bulk tissue transcriptome at DIV1. The transcriptome gradually shifted away from this early state and became stable by DIV6. **B** Gene set enrichment analysis of driver genes for each principal component was performed to identify salient biology described by each axis. − Log_10_[adj p-val] for enriched Kegg pathways is shown. **C** Hierarchical clustering of the full data set revealed a subset of genes whose differential expression shifted from robust increases early in culture (DIV1–4) to decreases later in culture (≥ DIV6). Manhattan hierarchical distances are shown to the right of full heat map and further highlight the differences in early vs. late transcriptomes. Scales for each heat map shown underneath and indicate Z-scores derived from Log_2_[Fold Change]. **D** Gene panels for specific aspects of the overall neuroinflammatory signature. Disease associated microglial gene panel indicates strong DAM2 state. Astrocytes were broadly activated and existed in both the neurotoxic (A1) and neuroprotective (A2) state. Complement C3 was robustly increased across all time points, however NLRP3 was modestly reduced. Expression of neurotransmitter receptor genes across all transmitter classes was robustly decreased. Scales for each heat map shown to the side and indicate Z-scores derived from Log_2_[Fold Change]

Shifts in microglia to a disease-like state in organotypic brain slice cultures indicate ongoing responses to the initial trauma of slice creation. Transcriptomes from bulk tissue preparations, which encompass all cell types including microglia, were analyzed using NanoString technology to assess global shifts in gene expression. Similar to what was observed in purified microglia, bulk transcriptomes from tissue after 1 day in culture exhibited robust changes when assessed via principal component analysis that resolved after 3 days in culture (Fig. 2A). Bulk transcriptomes appeared to reach a steady-state after 6 days in culture, which was confirmed using k-means clustering (Fig. 2A). Gene set enrichment analysis (GSEA) indicated that the changes described by principal component 1 were mostly driven by synaptic biology, whereas principal component 2 was driven by inflammatory and apoptotic/survival pathways (Fig. 2B). These observations are consistent with early biological responses to injury and trauma (DIV1) resolving into an ongoing chronic inflammatory state (≥ DIV6). Hierarchical clustering was used to visualize and orthogonally confirm the transcriptional patterns characterized using principal component analysis. Differential gene expression patterns measured at DIV1 were the most distinct from all other days in culture (Fig. 2C). As initially observed with principal component analysis, the transcriptomes from DIV6-14 exhibited a high level of similarity (Fig. 2C). A subset of genes involved in a broad set of processes exhibited a shift from initial upregulation on DIV1 to downregulation by DIV6 (Fig. 2C). Further assessment of functional state of slice cultures using focused gene panels indicated a strong shift into the microglial DAM2 disease state [23], broad activation of astrocytes [49–51], robust activation of complement C3, mild engagement of the inflammasome, and a robust and enduring suppression of synaptic function genes (Fig. 2D).

**Fig. 3:**
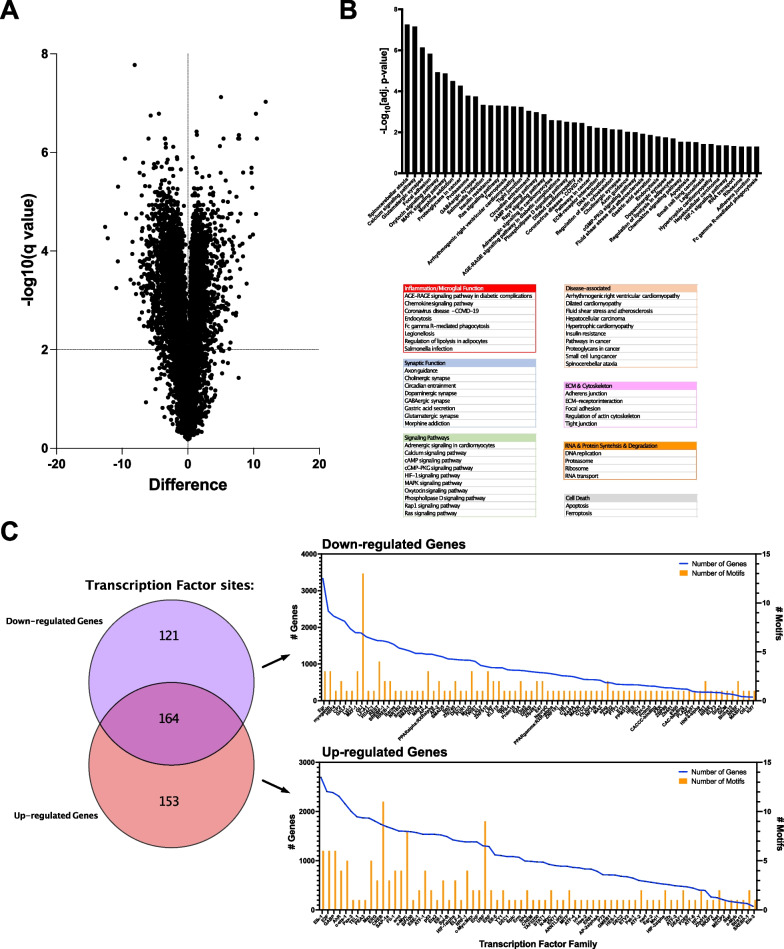
RNA-seq analysis of bulk transcriptomes reveal transcriptional regulation associated with inflammation, synaptic function, and disease biology. RNA-seq analysis of bulk tissue transcriptome was performed at DIV6. **A** Volcano plot of differential gene expression. **B** Gene set enrichment analysis of significantly dysregulated genes was performed to identify salient biology. − Log_10_[adj p-val] for enriched Kegg pathways is shown. Significant functional pathways are grouped below the histogram to highlight commonalities in dysregulated biology. **C** Transcription factor enrichment was assessed in genes that were significantly differentially expressed. A summary of the transcription factors, number of distinct motifs, and the number of genes that contain these motifs in their promoter region is shown to the right of the summary Venn diagram

Transcriptomes from isolated microglia and bulk tissue indicate an acute transitional phase that occurs around DIV1 and resolves by DIV6, at which point the cultures are stable through DIV14. To further understand the changes that occur once slice cultures enter a stable neuroinflammatory state, bulk tissue transcriptomes were characterized using RNA-seq. Gene set enrichment analysis indicated that the pattern of differential gene expression was associated with functional pathways of inflammation, synaptic function, and disease (Fig. 3). To determine which cell types contribute to the overall pattern of differential gene expression, transcription factor sites that were significantly enriched in differentially expressed genes (Fig. 3) were mapped to cell types based on published gene expression databases for mouse brain [52]. Transcription factors that mediate increases in gene expression, exhibited the highest expression in astrocytes, oligodendrocyte precursor cells, microglia, and neurons (Additional file 1: Fig. S1A). Expression of transcription factors that mediated decreases in gene expression was highest in astrocytes, oligodendrocyte precursor cells, and neurons (Additional file 1: Fig. S1B). Additionally, mature, myelinating oligodendrocytes appeared to contribute little the differential gene expression in slice cultures (Additional file 1: Fig. S1A, B). Unlike other cell types implicated by this analysis, microglia were associated with transcription factors that primarily contributed to increased gene expression and not to decreases in gene expression, suggesting that microglia are a primary driver of the changes in other cell types.

### Microglia are necessary for astrocyte activation

Depletion of microglia using well characterized CSF1R inhibitors have been useful in identifying inflammatory and/or degenerative processes that are modulated or dependent upon these cells [53–59]. Mice were fed PLX3397, a potent CSF1R tyrosine kinase inhibitor, to deplete microglia from the CNS. Immunohistochemical analysis of cortex confirmed that microglia were completely depleted from CNS after 7 days of PLX3397 dosing, after which the depletion was stable for at least 14 days (Additional file 2: Fig. S2A). Attempts to culture primary microglia from mice that were dosed with PLX3397 failed (Additional file 2: Fig. S2B), indicating that any residual IBA1 immunostaining was associated with dead or dying microglia.

**Fig. 4:**
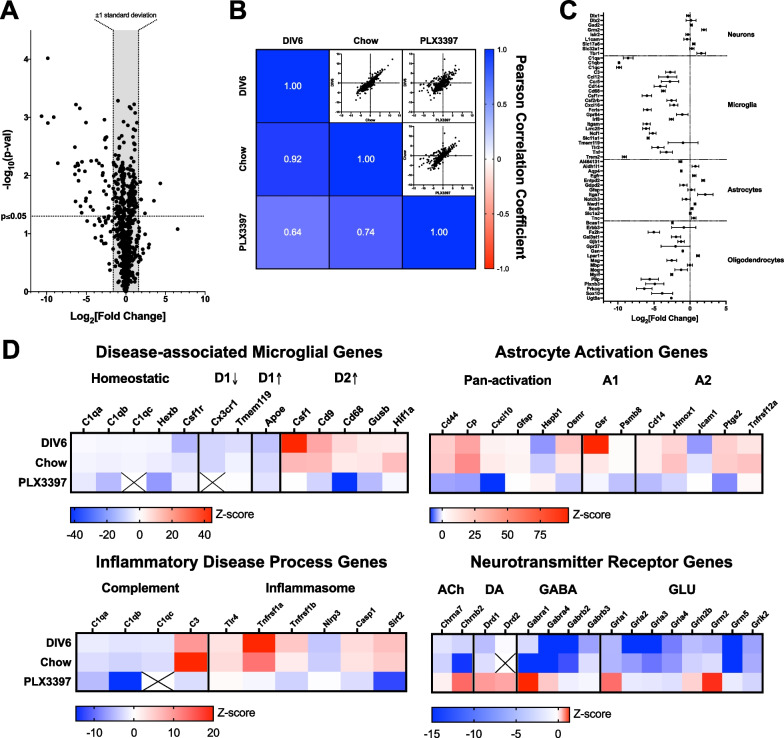
Depletion of microglia prevents activation of astrocytes. Mice were fed the CSF1R inhibitor PLX3397 in chow for 10 days prior to creation of organotypic brain slice cultures. Transcriptomic analysis was performed using NanoString from tissue harvested at DIV6. **A** Volcano plot of differential gene expression in PLX3397-fed mice. Note the robust decreases observed across a number of genes. **B** Pearson correlation matrix indicates a high correlation (*r* = 0.92) in mice that received standard chow relative to the previous DIV6 transcriptome presented in Fig. 2. Transcriptome derived from mice that were fed PLX3397 exhibits much lower correlations to both control transcriptomes. **C** Expression of genes that specific for distinct cell types indicate a decrease in both microglial and oligodendrocyte gene expression. **D** Organotypic brain slice cultures derived from PLX3397-fed mice do not exhibit transcriptomic changes that indicate a microglial disease state, do not show evidence for activation of astrocytes, do not exhibit increases in complement or inflammasome, and do not exhibit widespread decreases in neurotransmitter receptor genes. Scales for each heat map shown underneath and indicate Z-scores derived from Log_2_[Fold Change]. X represents genes that were not detectable

Transcriptomic analysis of brain tissue from mice treated with PLX3397 indicated robust decreases in gene expression (Fig. 4A) and an overall reduction in alignment with transcriptomes observed previously (Fig. 4B). Using a panel of genes that are expressed in specific cell types, microglial depletion resulted in robust loss of microglia-specific genes (Fig. 4C). Similar to previous studies [55], PLX3397 treatment was also associated with a loss of oligodendrocyte genes (Fig. 4C). Importantly, treatment with PLX3397 did not affect expression of neuron or astrocyte-specific genes, indicating these cell populations remained intact and were not directly affected by PLX3397 treatment. As expected, microglial depletion was associated with loss of the microglial DAM signature (Fig. 4D), which is likely due to the absence of these cells. Of note, transcriptional signatures of astrocyte activation were also lost in the absence of microglia (Fig. 4D), consistent with previous observations of the necessity of microglia for the activation of astrocytes [49, 60–62]. Microglial depletion also prevented perturbations in complement and inflammasome-related genes, indicating broader suppression of the overall inflammatory axis (Fig. 4D). Lastly, microglial depletion largely prevented the suppression of neurotransmitter genes and for some genes, expression was promoted (Fig. 4D).

### Longitudinal pattern of protein secretion

**Fig. 5:**
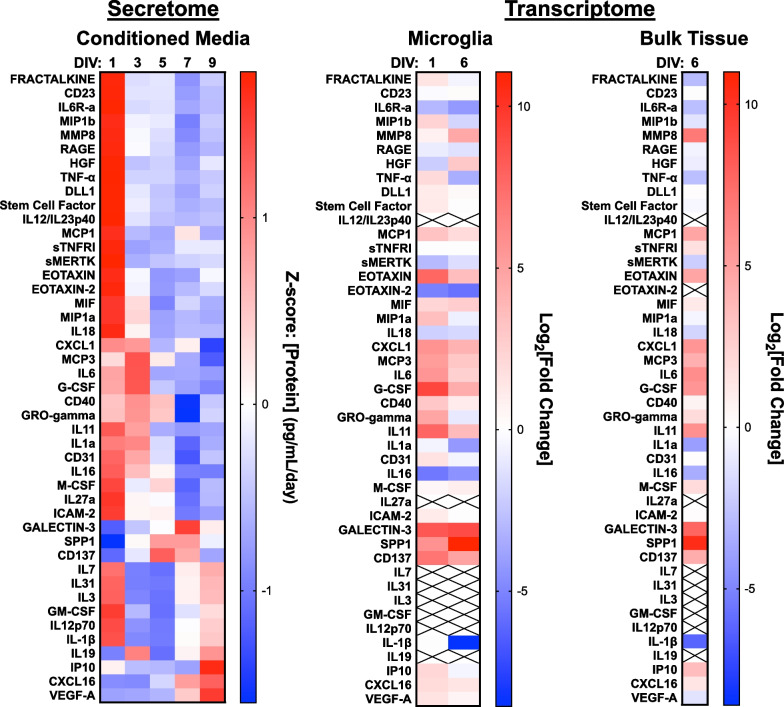
Differential protein secretion reveals pattern of inflammatory mediators. Soluble proteins relevant to inflammation were quantified from conditioned media using the FirePlex multiplexed assay. Heat map indicates Z-scores of protein levels (ng/mL/day) measured in media over time in culture. For reference, transcriptomes from isolated microglia and bulk tissue are shown to the right of the secretomic data. X represents proteins that were not detectable

Microglia communicate with neighboring cells by several mechanisms, including through secreted signaling molecules. A survey of changes in secreted proteins relevant to inflammation was performed using the multiplexed FirePlex assay platform. A number of proteins were observed to undergo distinct patterns of temporal regulation, revealed by hierarchical clustering, which was consistent with transcriptomic observations. A considerable proportion of secreted inflammatory proteins exhibited a significant and robust increase in secretion on DIV1, followed by suppression of secretion up to DIV 9 (Fig. 5). The proteins in this group included neuron–microglia mediators such as fractalkine and stem cell factor; chemoattractants like eotaxin, MCP1, and MIP-1α/β; and pro-inflammatory mediators such as TNF-α, MIF, and IL-12p40 (Fig. 5). A second, distinct group of proteins exhibited robust increases in secretion during the period where slice cultures transitioned to a stable neuroinflammatory signature as assessed trancriptionally. These proteins included Galectin-3, a known pro-inflammatory modulator that acts through Trem2 [63], SPP1/osteopontin, which is involved in wound repair and has been recently implicated in AD risk and identified as one of a multitude of molecular markers for activated response microglia [64], and CD137, which is a pro-inflammatory cytokine that is implicated in apoptosis of oligodendrocytes [65]. A final group of secreted proteins exhibit a late increase in secretion and are generally associated with cell death and senescence such as IL-1β and IP10, and proteins involved in microglial proliferation like GM-CSF. While not included in the proteomics panel used in these analyses, other senescence genes such as p16 (cdkn2a) and p21 (cdkn1a) exhibited increases in expression in isolated microglia and bulk tissue RNA-seq analyses further supporting cellular senescence as an important domain of biology contributing to the inflammatory environment. This pattern suggests an upregulation of Trem2-dependent processes in microglia [66, 67] and is consistent with the transcriptomic signatures in microglia (Fig. 1D) and bulk tissue (Fig. 2D).

Analysis of the microglial transcriptomic data indicated that changes in the transcriptome occurred 1–3 days prior to changes in secreted proteins, where transcripts could be detected (Fig. 5). Most transcriptomic changes observed in purified microglia were also observed in the transcriptomes isolated from bulk tissue at DIV6 (Fig. 5). Collectively, these results confirm that transcriptomic changes are a leading index of changes in protein expression.

### Alignment of transcriptomic signatures in murine cultures with human disease

**Fig. 6:**
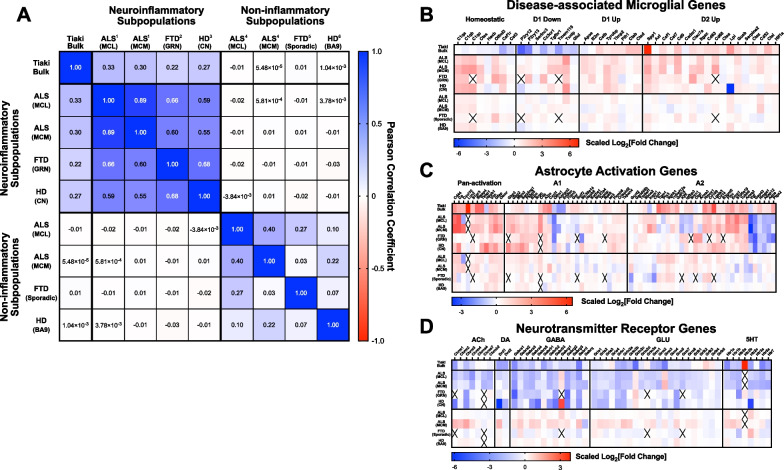
Slice cultures recapitulate salient features of neuroinflammatory disease biology observed in patients. Publicly available transcriptomic data from patients with either ALS, FTD or HD were assessed for similarity to the transcriptomic platform data. **A** Pearson correlation (r) matrix indicates a high degree of correlation in transcriptomes from subsets of patients in each disease group with inflammatory biology. Non-inflammatory populations, or unaffected brain regions (HD) exhibit no correlation to platform or neuroinflammatory patient populations. **B** Conservation of the DAM gene signature between platform and patient data. Control patient populations show little to no change in these genes. **C** Conservation of astrocyte activation signature between platform and patient data. Control patient populations show little to no change in these genes. **D** Conservation of loss of neurotransmitter receptor gene expression between platform and patient data. Control patient populations show little to no change in these genes. Superscripts indicate data sources: 1 = [5] glial subpopulation, 2 = GSE13162 grn mutation carriers, 3 = GSE3790 caudate nucleus samples, 4 = [5] Other patient subpopulations, 2 = GSE13162 Sporadic FTD patients, 3 = GSE3790 Broca’s Area 9 samples

The increased accessibility of machine learning-enabled analytical tools combined with deep sequencing transcriptomics has facilitated the stratification of patient populations with various neurodegenerative disorders. Recent studies have identified subpopulations of patients [4, 5] that exhibit robust dysregulation of glial cells and evidence of neuroinflammation. Comparison of the bulk tissue transcriptome from slice cultures to the published bulk transcriptome of the neuroinflammatory ALS patient subpopulation indicated significant alignment with transcriptomes from the medial and lateral cortices (Fig. 6A). No alignment was observed in the same brain regions when compared to the other subtypes identified by Tam OH*, *et al*.* [5] (Fig. 6A). Alignment was also seen in patients with frontotemporal dementia that harbored a mutation in the *grn* gene, but not in patients with sporadic disease [68] In patients with Huntington’s disease, alignment was observed to transcriptomes from caudate nucleus but not Brodmann’s area 9, indicating specificity to processes related to protein aggregation and active neurodegeneration [69, 70].

Using focused gene panels, transcriptomic alignments were further characterized. Bulk tissue transcriptomes from patient subpopulations exhibited increases in gene expression suggestive of overall microglial proliferation, including expansions in disease-associated microglia (Fig. 6B). Strong alignments were seen with respect to astrocyte activation (Fig. 6C) and neurotransmitter receptor expression loss (Fig. 6D). Additional gene panels were used to confirm rodent brain slice cultures recapitulated human gene signatures. Good alignment was observed when using either the ‘HAM’ [24] (Additional file 3: Fig. S3A) or the ‘LDAM’ (lipid-droplet-accumulating microglia) [25] (Additional file 3: Fig. S3B) gene signatures. There was modest alignment with the senescence-associated ‘SenoMayo’ [71] gene signature (Additional file 3: Fig. S3C). Collectively, these observations confirm murine cortical brain slice cultures recapitulate human disease-relevant neuroinflammatory biology.

### Brain slice cultures are responsive to pharmacological modulators of microglia

**Fig. 7:**
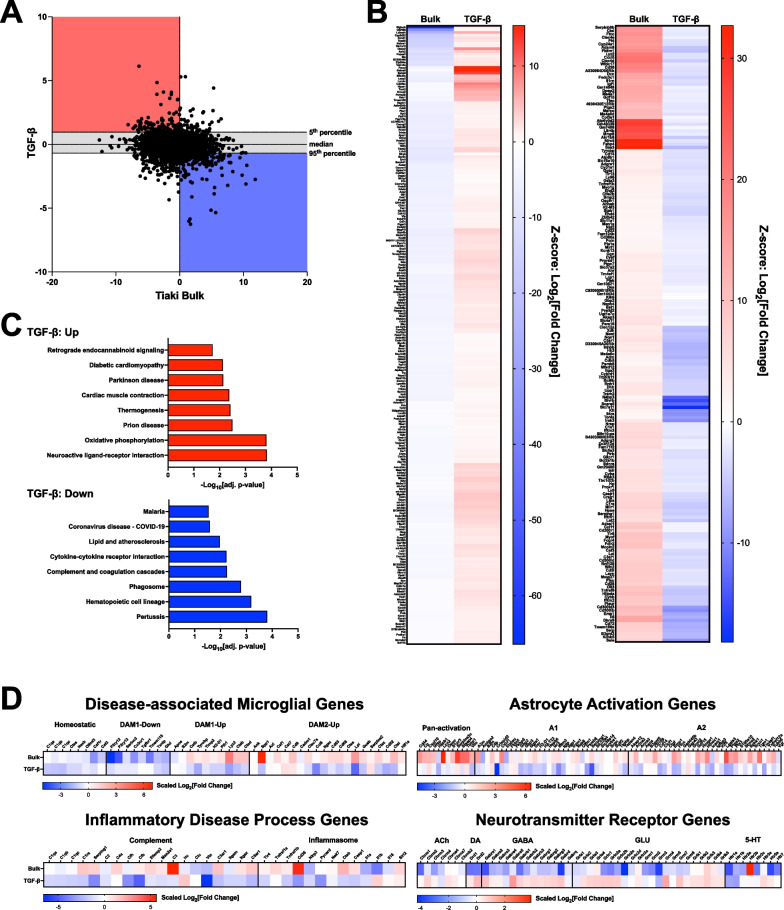
TGF-β2 mitigates neuroinflammatory disease biology in slice cultures. RNA-seq analysis of bulk tissue derived from organotypic brain slice cultures treated with TGF-β2. **A** Comparison of transcriptomes from slices treated with TGF-β2 vs. naïve slices, shown as log2 fold change. Highlighted are regions where TGF-β2 increased genes that are decreased in naïve slices (red), and where TGF-β2 decreased genes that are increased in naïve slices (blue). **B** Heat maps depicting hierarchically clustered differential gene expression of ‘red’ (left) and ‘blue’ (right) genes. This visualization highlights the robust reversals induced by TGF-β2. **C** Gene set enrichment analysis identified Kegg pathways that were significantly enriched in ‘red’ and ‘blue’ gene groups. **D** TGF-β2 robustly reversed gene expression related to disease-associated microglia, astrocyte activation, complement and inflammasome pathways, and neurotransmitter receptors. Scales for each heat map shown underneath and indicate Z-scores derived from Log_2_[Fold Change]

Quantitative responsivity to relevant pharmacological agents is required for any platform to be considered fit-for-purpose in drug discovery. Transforming growth factor beta (TGF-β) has been consistently shown to be a potent immunomodulator that drives microglia towards a more supportive and/or homeostatic state [72, 73]. We assessed the effect of recombinant TGF-β2 on neuroinflammation via RNA-seq transcriptomic analysis of bulk tissue (Fig. 7A). Our initial analysis focused on differential gene expression induced by TGF-β2 treatment that were oppositional to the differential gene expression observed in untreated slices (Fig. 7B). Gene set enrichment analysis indicated that the genes increased by TGF-β2 treatment were generally associated with synaptic function, while genes decreased by TGF-β2 treatment were associated with pathways describing pro-inflammatory processes (Fig. 7C). Further assessment of the patterns of differential gene expression induced by TGF-β2 treatment using defined gene panels indicated that TGF-β2 suppressed gene expression associated with the DAM state and astrocyte activation (Fig. 7D). TGF-β2 also broadly suppressed genes associated with the complement pathway and inflammasome (Fig. 7D). Lastly, TGF-β2 increased genes associated with synaptic function (Fig. 7D). Collectively, this pattern of differential gene expression is consistent with the documented effects of TGF-β on microglial functional state and confirms that the cortical brain slice cultures are appropriately responsive to pharmacological intervention.

### Patient-relevant neuroinflammation is pharmacologically unique

Demonstration that the chronic neuroinflammatory state could be modulated pharmacologically with recombinant TGF-β indicated that the organotypic brain slice system could be used to screen for targets that could modulate this disease biology. Several classes of anti-inflammatories have been evaluated clinically or have been postulated to be beneficial based on preclinical studies. The platform was initially used to survey a broad range of targets implicated as generally anti-inflammatory: glucocorticoid receptors, COX-2, dihydrofolate reductase, and the NLRP3 inflammasome. The agents used to assess these targets, dexamethasone, rofecoxib, methotrexate, and CRID3, respectively, were well characterized small molecules that are generally accepted to potently modulate the primary targets of interest. Importantly, all agents were applied to the cultures at DIV5, a time point where the neuroinflammatory state is fully developed and stable (Figs. 1, 2). For these studies, the NanoString Neuropathology panel was used to do a focused survey of the transcriptome relevant to neuroinflammation and neurodegenerative disease.

**Fig. 8:**
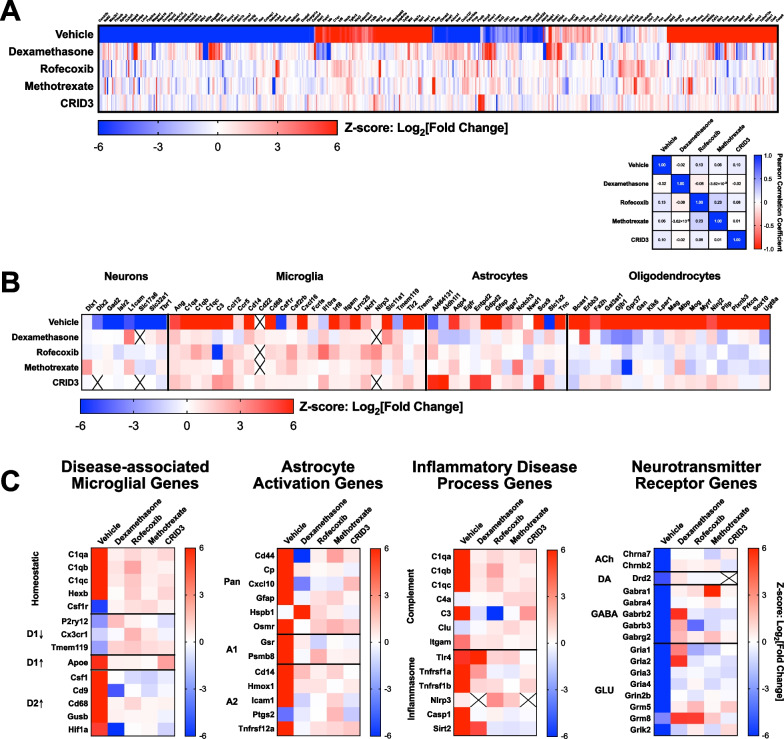
Canonical anti-inflammatory agents do not affect disease-relevant neuroinflammation. NanoString analysis of bulk tissue transcriptomes after treatment with anti-inflammatory agents. **A** Hierarchical clustering of full transcriptomes highlight a lack of a consistent pattern of reversal by any of the agents assessed. Pearson correlation matrix of transcriptomic data confirm lack of robust reversal of chronic neuroinflammatory state. **B** Analysis of genes that exhibit cell type-specific expression indicates general increases in microglia and astrocyte-associated transcription. **C** Heat maps of gene panels for specific domains of biology indicate modest effects on microglia, but either no effects or exacerbation of disease state with respect to gene expression related to astrocyte activation, inflammatory processes, and neurotransmitter receptors. Heat maps in this figure indicate Z-scores derived from Log_2_[Fold Change]. Z-scores were capped at ± 6 in Panel C to facilitate visualization

Hierarchical clustering of the full transcriptomic data set indicated no coherent reversal of the neuroinflammatory state, with Pearson correlation analysis indicating modest exacerbation (Fig. 8A). These results were unexpected and indicated that biological pathways implicated in inflammatory processes in peripheral compartments were not key drivers of microglia-centric chronic inflammation. Cell type-specific gene expression indicated that all treatments evaluated increased expression of microglial genes, and modestly increased astrocyte gene expression (Fig. 8B). All treatments assessed tended to promote microglial gene expression in general, indicating no clear modulation of these cells back to homeostasis. In addition, these treatments also promoted activation of astrocytes, activation of the NLRP3 inflammasome, and had no effects on neurotransmitter receptor expression (Fig. 8C). Taken together, these data indicate conventional anti-inflammatory targets are not sufficient to reverse the chronic neuroinflammatory state associated with neurodegenerative disease.

### Identification of PTPN11 (SHP2) as a novel neuroinflammation target

The failure of conventional anti-inflammatory targets, such as COX-2, to reverse the system-wide neuroinflammatory signature in our organotypic brain slice culture system mirrored what has been observed in clinical studies of these agents [74]. Thus, the effects observed with these agents provide validation of the negative predictive value of data from the slice culture platform. The transcriptomic data from microglia were further mined to find a novel, potent, druggable target that could reverse the neuroinflammatory signature. We hypothesized that receptors or families of receptors and their associated signaling apparatus that were enriched or specifically expressed on microglia that also exhibited significant dysregulation coincident with chronic neuroinflammation would represent the best targets for small molecule interventions.

**Fig. 9:**
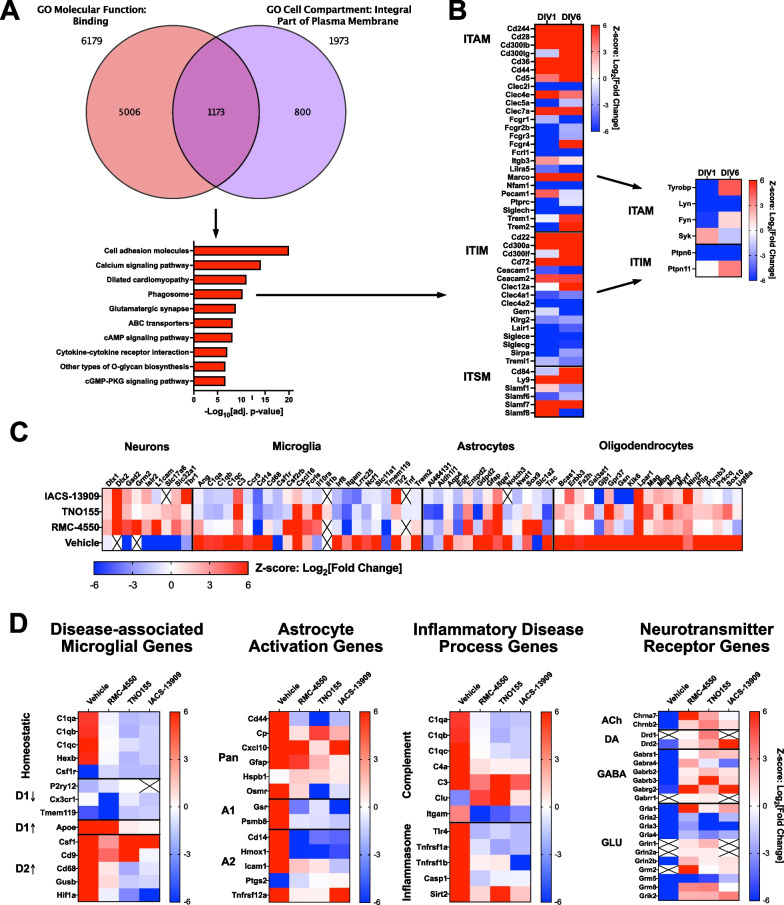
Identification and validation of PTPN11 (SHP-2) as a novel target to reverse chronic neuroinflammation. **A** Venn diagram of significant DEGs that were associated with the GO term(s) of “Molecular Function: Binding” and “Cell Compartment: Integral Part of Plasma Membrane”. 1,173 genes identified by this analysis were then analyzed via GSEA for Kegg pathways. Significant pathways and associated adjusted p-values indicated in histogram. **B** Heat map of differential gene expression of phagocytosis receptor genes, grouped by whether the protein encoded by the gene signals via ITAM, ITIM, or ITSM. Inset indicates differential expression of genes for signaling molecules associated with ITAM and ITIM receptors. **C** Analysis of genes that exhibit cell type-specific expression indicates increases in neuron-associated transcription and decreases in microglial and astrocyte transcription in response to inhibitors of PTPN11. **D** Heat maps of gene panels for specific domains of biology indicate suppression of disease-associated microglia genes, astrocyte activation genes, and genes associated with inflammatory processes. Genes associated with neurotransmitter receptors were increased

Gene set enrichment analysis for GO terms using the microglial differentially expressed genes as the dataset was performed and confirmed that ‘integral part of plasma membrane’ (cellular compartment) and ‘binding’ (cellular function) were both significantly enriched (FDR ≤ 0.05), indicating that our initial strategy was feasible. Using these terms, we identified a subset of differentially expressed genes in microglia (Fig. 9A). Performing GSEA on these genes for Kegg pathways indicated several pathways relevant to microglial function were enriched in the GO term overlaps (Fig. 9A). The most significantly enriched pathway that is associated with current drug discovery efforts, suggesting overall druggability, was the phagosome pathway, which describes processes involved in phagocytosis (Fig. 9A). Modulation of various phagocytosis receptors are currently under investigation as potential therapeutic strategies for modulation of neuroinflammation. We broadly surveyed the transcriptional dysregulation of phagocytosis receptors that signal through immunoreceptor tyrosine activation motifs (ITAM), immunoreceptor tyrosine inhibition motifs (ITIM), or immunoreceptor tyrosine switching motifs (ITSM). There was an increase in the expression of a subset of phagocytosis receptors that signal through ITAM and thus, promote phagocytosis activity and inflammation resolution in microglia (Fig. 9B). Not all ITAM-associated receptors contain ITAM domains in their cytoplasmic tails, and a number of receptors rely on adapter proteins like FcRɣ or Tyrobp (DAP12) to mediate ITAM signaling [75]. There was a robust suppression of the major microglia FcRɣ genes combined with a modest suppression of Syk, suggesting an overall attenuation of ITAM signaling despite an increase in some of the ITAM receptors (Fig. 9B). In addition, the expression of genes for a subset of ITIM-containing receptors was robustly increased along with the primary signaling molecule for these receptors, PTPN11 (Fig. 9B). Gene expression for a number of receptors with switching motifs (ITSM) was also observed, however none of the genes for the docking proteins that mediate signaling for these receptors was detected in our study with the exception of PTPN6 and PTPN11, suggesting ITSM-containing receptors are primarily inhibitory in mouse microglia (Fig. 9A). The expression of Ptpn11 was significantly increased in microglia on DIV6, indicating an increase in signaling oppositional to ITAM-mediated signaling (Fig. 9B). PTPN11 is currently under active development for oncology indications due to its direct association with PD-1 via ITIM and ITSM domains [76]. We leveraged several different specific, small molecule allosteric inhibitors to investigate whether inhibition of PTPN11 was beneficial for resolving chronic neuroinflammation.

Three structurally distinct allosteric inhibitors of PTPN11 were used to assess the potential role of SHP2 in mitigating neuroinflammation: RMC-4550 [77], TNO155 [78], and IACS-13909 [79]. Dose-dependent modulation of gene expression was observed after exposure of organotypic slices to either RMC-4550, TNO155 or IACS-13909 (Additional file 4: Fig. S4). Comparison of the effects of highest dose of all PTPN11 inhibitors evaluated indicated robust reversal of the neuroinflammatory signature. All compounds exhibited strong effects to promote expression of neuronal genes, with modest effects on microglial and astrocyte genes (Fig. 9C). All compounds exhibited beneficial effects when benchmarked using the DAM, astrocyte activation, inflammatory disease, and neurotransmitter receptor gene panels (Fig. 9D). PTPN11 inhibition was associated with a suppression of DAM2 pattern of gene expression in microglia, suppression of astrocyte activation, suppression of inflammasome pathway genes, and promotion of neurotransmitter receptor genes (Fig. 9D). Interestingly, the neurotransmitter receptor genes that exhibit decreases in response to PTPN11 inhibition (Fig. 9D) are known to be enriched in non-neuronal cells including astrocytes and oligodendrocytes [52], highlighting the cellular specificity of the pro-synaptic effect of PTPN11 inhibition.

## Discussion

Classical translational models for neurodegenerative research have focused on recapitulation of pathology, but not necessarily the underlying biological processes, that drive the cells and tissues to create those pathologies. For example, there are numerous mouse lines that model the appearance of β-amyloidosis and parenchymal plaques that are observed in patients with Alzheimer’s disease, but most of these models accomplish this through supra-physiological expression of these proteins rather than faithfully modeling the biological disruptions that occur in patients [80]. These types of models may be sufficient for clinical translation of therapeutic strategies focused solely on pathology, but lack utility for other mechanisms. There is a critical unmet need for easily accessible, translational models of patient-relevant disease biology.

Drug discovery for CNS indications has benefited greatly from the implementation of machine learning into nearly every facet of the process [81]. One of the most transformative uses of bioinformatics is the identification of distinct molecular subtypes of patients with CNS diseases such as Alzheimer’s disease [4, 82], amyotrophic lateral sclerosis [5], Parkinson’s disease [83], and schizophrenia [84]. These analyses have highlighted the importance of neuroinflammation, innate immune system function, and microglia specifically as potential drivers of disease pathogenesis in specific subgroups of patients. To understand the causal relationship more fully between CNS dysfunction and neuroinflammation, molecular targets that robustly modulate microglial functional state and mitigate neuroinflammatory biology must be identified and prosecuted. Tools that enable faithful translation of patient-relevant neuroinflammatory disease biology in a setting that is accessible in a conventional drug discovery environment are needed.

Herein we describe an organotypic brain slice culture system that recapitulates patient-relevant neuroinflammatory disease biology. Conservation of the neuroinflammatory signature across diseases and even species reflects the high degree of conservation of the innate immune system itself and its role in maintenance of overall CNS homeostasis. The differences in progression and end-stage pathology associated with neurodegenerative diseases likely reflect differences in brain regions and neural circuits initially impacted by innate immune system dysfunction. This neuroinflammatory platform leverages this conservation and is built using brain tissue derived from mature mouse brain, making it highly accessible and amenable to genetic manipulation. Depletion of microglia not only prevents the manifestation of neuroinflammation, but astrocyte activation was also absent, indicating that microglial dysfunction is necessary for complete manifestation of this biology at a systems level. Consistent with this observation, transcription factor enrichment analysis of bulk tissue transcriptomics also identified changes in microglia as likely drivers of the overall patterns in differential gene expression observed in bulk tissue.

One notable feature of the differential gene expression pattern observed in both patients and in slice cultures was the loss of expression of synaptic function genes. Depletion of microglia did not have the same effect, indicating an active involvement of dysfunctional microglia in the induction of this phenomenon. We also observed robust suppression of genes associated with myelination and oligodendrocyte maturation and function in the context of microglial depletion and acute tissue trauma, consistent with previous reports [55], suggesting a broad role of microglia in maintenance of neural network function.

The interplay between microglia, oligodendrocytes, and overall myelination is of keen interest in many neurodegenerative diseases [85]. In our studies, PLX3397 treatment was associated with the expected loss of microglia gene expression, but also oligodendrocyte gene expression. PLX3397 is a semi-selective tyrosine kinase inhibitor that preferentially inhibits tyrosine kinase signaling via CSF1R [86]. Loss of oligodendrocytes and myelination has been demonstrated in the absence of microglia, however the pharmacologic interventions with PLX3397 to reduce microglia in these studies are not specific enough to draw definitive conclusions around the interplay between microglia and myelination [55].

To assess the utility of the platform for screening of pharmacological agents, a number of treatments were assessed. TGF-β, a potent homeostasis-inducing modulator of microglial function, reversed every aspect of patient-relevant neuroinflammatory disease biology. This is consistent with dysfunctional microglia being the key cellular drivers of neurodegenerative disease and highlights the potent and rapid restorative effects microglia can exert when in a more neuroprotective activation state.

One important aspect of a discovery platform is the capacity for negative prediction—the ability to definitively predict a negative outcome in subsequent studies. The well characterized anti-inflammatory agents selected for profiling in the platform as part of the experiments in Fig. 8 were chosen because they all have demonstrated anti-inflammatory activity in peripheral compartments. None of these agents exhibited significant efficacy to reverse the neuroinflammatory signature in the platform. Corticosteroids like dexamethasone potently suppress inflammatory states in peripheral immune cells but carry severe liabilities with chronic treatment [87]. The minimal benefit observed with dexamethasone in the platform suggests the risks of this therapeutic strategy outweigh any potential benefit. Similarly, COX-2 inhibitors have been assessed in patients for efficacy in Alzheimer’s disease and have failed [88, 89], mirroring observations reported in this study. Anti-rheumatoid therapeutics have been reported to have potential benefit in Alzheimer’s patients [90] however, we see no benefit of methotrexate in the platform, suggesting this specific mechanism of action as not optimal for chronic neuroinflammation. Finally, the failure of CRID3, a NLRP3 inflammasome inhibitor, was consistent with our transcriptomic analyses indicating no clear signature of inflammasome activation in the platform, and also our analyses of human transcriptomes that did not exhibit a strong signature of NLRP3 inflammasome activation. Moreover, measures of IL-1β indicate that the highest levels of secretion occur from DIV0-1 in the platform (Fig. 5), suggesting that the NLRP3 inflammasome contributes to induction of chronic neuroinflammation but not maintenance of the disease state. This observation suggests the potential utility of modulating the NLRP3 inflammasome earlier in the development of chronic neuroinflammation as a potential therapeutic strategy.

Leveraging the gene signatures revealed by the platform and validated against patient transcriptomic datasets, dysregulation of phagocytosis and the phagosome was identified as a major contributor to the overall transcriptomic signature of neuroinflammation, consistent with previous studies characterizing the role of individual components of this system in neurodegeneration [91]. A number of preclinical and clinical programs are exploring the efficacy of modulating individual phagocytosis receptors in mitigating neurodegenerative diseases, however our transcriptomic analyses indicate that gene expression for a number of different phagocytosis receptors is highly dysregulated. Focusing on the signaling nodes engaged by these receptors, we observed an overall downregulation of ITAM signaling capacity, and an increase in expression of PTPN11, a phosphatase involved in inhibiting ITAM signaling through the protein tyrosine kinase SYK. Three structurally distinct allosteric inhibitors of PTPN11 reversed the transcriptomic signature of neuroinflammation, demonstrating the potential utility of SHP2 inhibition as an anti-neuroinflammatory agent.

Neuroinflammation is increasingly recognized as a key driver of CNS disease, including neurodegenerative and neuropsychiatric disease. These diseases are multifactorial and will likely require multi-targeted therapeutic strategies. The utility of platforms like the one described in this study is that disease biology is modeled at a systems level in a target-agnostic fashion, enabling comprehensive screens for therapeutic molecules in neuroinflammation and other domains of biology such as neuroprotection and synaptic enhancement. Given the focus on a systems biology approach, our platform also affords the opportunity for a multi-omics approach including proteomics and metabolomics, which would complement the transcriptomic approach described here. Future studies will explore the association between changes in the transcriptome and other omics to better understand how to interpret transcriptomics and further explore druggable nodes in the network of neurodegenerative disease biology.

In this study, we document the creation of a translational, systems biology model of patient-relevant neuroinflammatory biology associated with neurodegenerative disease. The platform described is accessible, sensitive to pharmacological manipulation, and provides unique insights into neuroinflammatory systems biology. Future studies leveraging this technology could exploit the potential of the platform to validate novel immunomodulatory targets, incorporate patient-relevant cells [27, 92] and support neuroinflammation drug discovery programs.

### Supplementary Information

**Additional file 1: Figure S1.** Mapping transcription factors significantly enriched in differentially expressed genes to specific cell types in the brain using single cell expression profiles. Heat maps of cell-type specific expression reported in Zhang Y*, *et al*.* [52] for transcription factors significantly enriched in bulk tissue RNA-seq transcriptome. Heat maps are shown for transcription factors enriched in genes that are increased (**A**) and decreased (**B**) at DIV6. A high average value indicates a greater predicted contribution of that cell type to the overall bulk transcriptome. Of note, myelinating oligodendrocytes are predicted to contribute relatively little to the bulk transcriptome. Additionally, microglia and newly formed oligodendrocytes are predicted to contribute differentially to increases vs. decreases in the transcriptome, with microglia exhibiting more increases predicted than decreases and vice versa.

**Additional file 2: Figure S2.** Depletion of viable microglia from CNS with PLX3397. **A** IBA-1 immunolabeling indicates complete loss of microglia from cortex of mice fed PLX3397. Shown are representative images from sections of mouse cortex. Scale bar indicates 100 µm and applies to all panels. **B** Microglia were isolated from cortex of brain from mice fed control chow or PLX3397 and placed into primary culture. Phase contrast images indicate no viable microglia were recovered from mice that were fed PLX3397. Scale bar indicates 100 µm and applies to all panels. **C** Analysis of gene expression of CD11-isolated cells using gene panels of cell type-specific enriched genes based on gene expression reported in Zhang Y*, *et al*.* [52]. Log2 expression data for the top 50 cell type-specific genes for each cell type was summed and plotted as a heatmap. Note the several fold enrichment of microglia-specific genes, indicative of a high-purity of microglia in **B**. Note that these gene expression data are from mice that were not fed PLX3397, indicating a likely underestimation of the purity of microglia in **B**.

**Additional file 3: Figure S3.** Brain slice cultures recapitulate human microglial disease signatures. **A** Heat map of genes from the Human AD microglia signature [24]. Conservation of the HAM gene signature between platform and patient data. Control patient populations show little to no change in these genes. **B** Heat map of genes from the Lipid-droplet-accumulating microglia signature [25]. Conservation of the LDAM gene signature between platform and patient data. Control patient populations show little to no change in these genes. **C** Heat map of genes from the human senescence-associated genes (“SenoMayo”) signature [71]. Senescence-associated signature was stronger in the patient data relative to control data. Heat maps indicate Scaled Log_2_[Fold Change]. ‘X’ indicates a gene that was undetected. Data sources are identical to Fig. 6.

**Additional file 4: Figure S4.** Dose-dependent modulation of gene expression by RMC-4550, TNO155, and IACS-13909. **A** RMC-4550 modulated gene expression in a dose-dependent manner. Volcano plot shows increasing number of genes with significant differential expression. Waterfall plot of genes that were significantly differentially expressed at the highest dose of RMC-4550 shows a dose-dependent increase in the area under the curve. Hierarchical clustering of all genes based on Z-scores of Log2-transformed fold changes illustrate dose-responsivity across all genes monitored. **B** TNO155 modulated gene expression in a dose-dependent manner. Volcano plot shows increasing number of genes with significant differential expression. Waterfall plot of genes that were significantly differentially expressed at the highest dose of TNO155 shows a dose-dependent increase in the area under the curve. Hierarchical clustering of all genes based on Z-scores of Log2-transformed fold changes illustrate dose-responsivity across all genes monitored. **C** IACS-13909 modulated gene expression in a dose-dependent manner. Volcano plot shows increasing number of genes with significant differential expression. Waterfall plot of genes that were significantly differentially expressed at the highest dose of IACS-13909 shows a dose-dependent increase in the area under the curve. Hierarchical clustering of all genes based on Z-scores of Log2-transformed fold changes illustrate dose-responsivity across all genes monitored.

## Data Availability

All data are available upon reasonable request. No unique materials were created as part of these studies.
