# Supplementary material for: Preclinical translational platform of neuroinflammatory disease biology relevant to neurodegenerative disease
Source: J Neuroinflammation. 2024 Jan 31;21:37. doi: 10.1186/s12974-024-03029-3 (PMC10832185; doi:10.1186/s12974-024-03029-3)

Supplemental Figure 1

A Transcription Factors: Up-regulated Genes

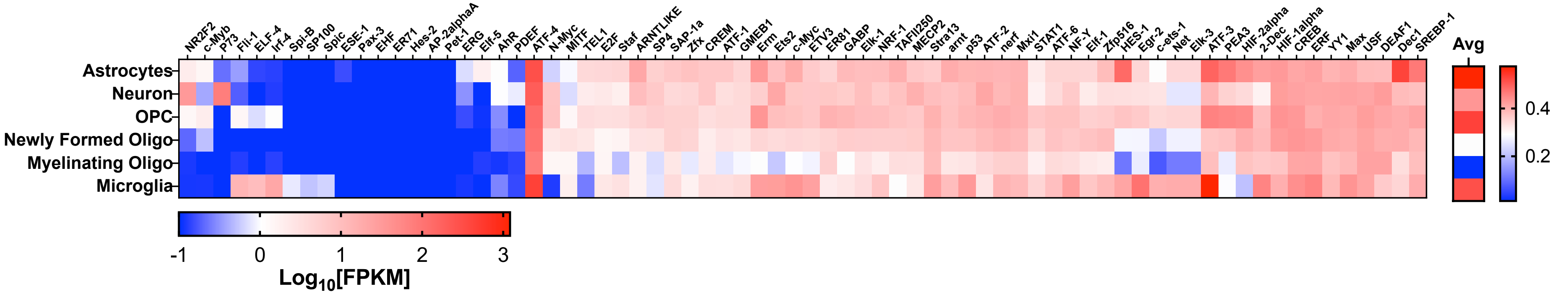

B Transcription Factors: Down-regulated Genes

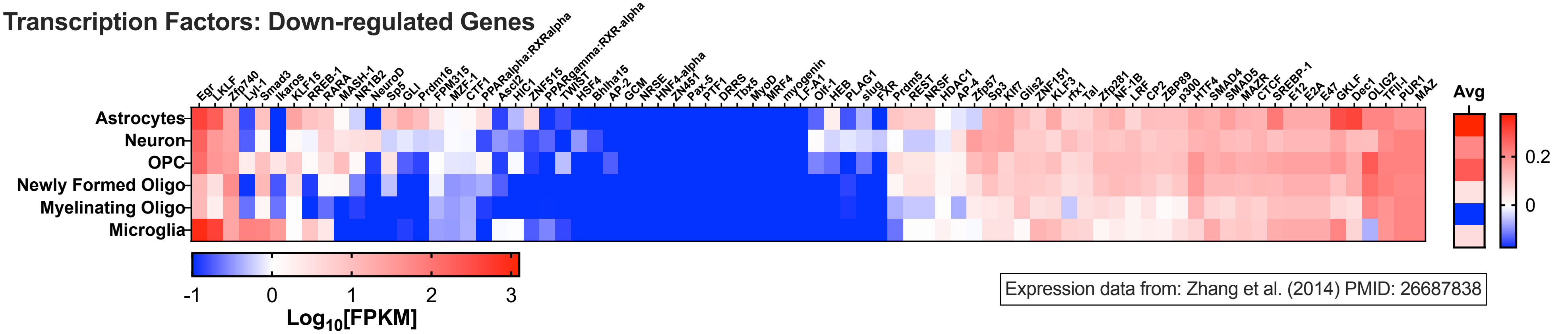

Supplement: Supplementary file 1 — Additional file 1: Figure S1. Mapping transcription factors significantly enriched in differentially expressed genes to specific cell types in the brain using single cell expression profiles. Heat maps of cell-type specific expression reported in Zhang Y, et al. [52] for transcription factors significantly enriched in bulk tissue RNA-seq transcriptome. Heat maps are shown for transcription factors enriched in genes that are increased (A) and decreased (B) at DIV6. A high average value indicates a greater predicted contribution of that cell type to the overall bulk transcriptome. Of note, myelinating oligodendrocytes are predicted to contribute relatively little to the bulk transcriptome. Additionally, microglia and newly formed oligodendrocytes are predicted to contribute differentially to increases vs. decreases in the transcriptome, with microglia exhibiting more increases predicted than decreases and vice versa. [file 12974_2024_3029_MOESM1_ESM.pdf]
