# Supplementary figures and images for: Preclinical translational platform of neuroinflammatory disease biology relevant to neurodegenerative disease
Source: J Neuroinflammation. 2024 Jan 31;21:37. doi: 10.1186/s12974-024-03029-3 (PMC10832185; doi:10.1186/s12974-024-03029-3)

# Supplemental Figure 2

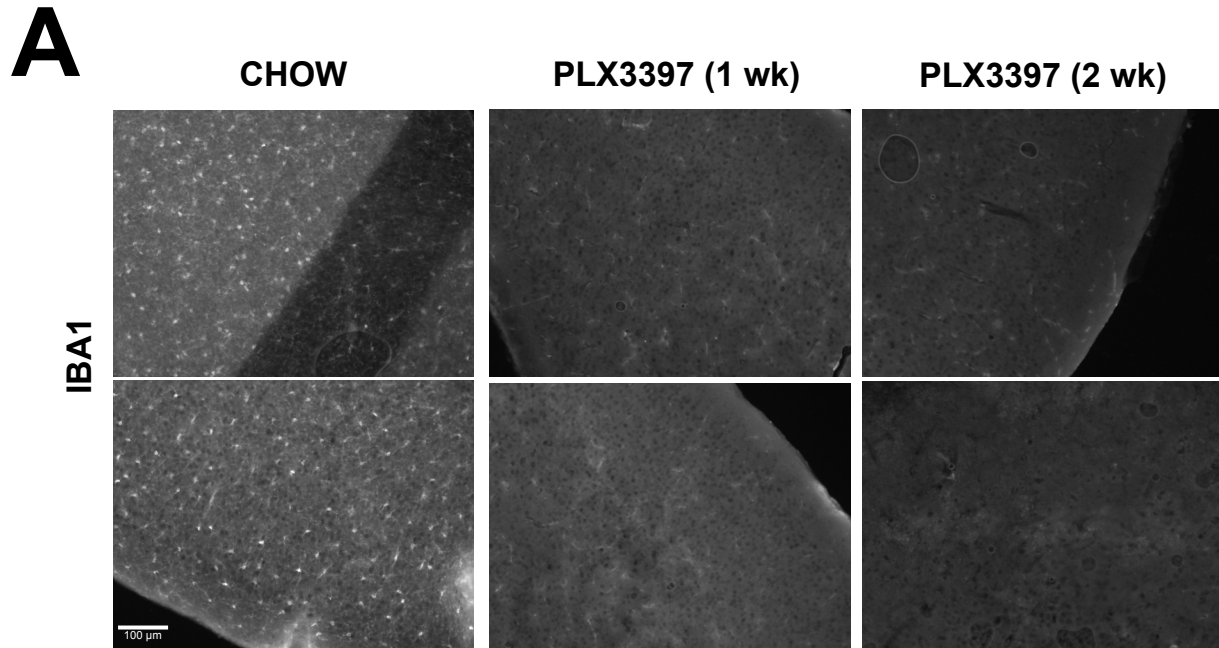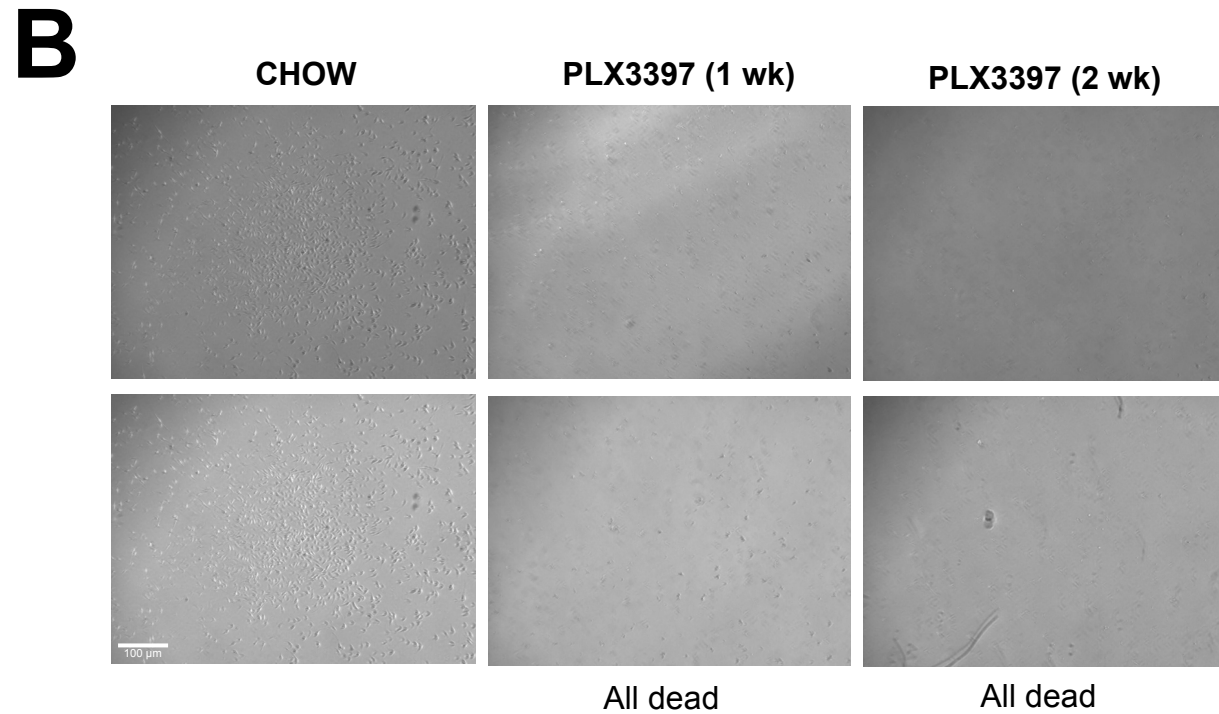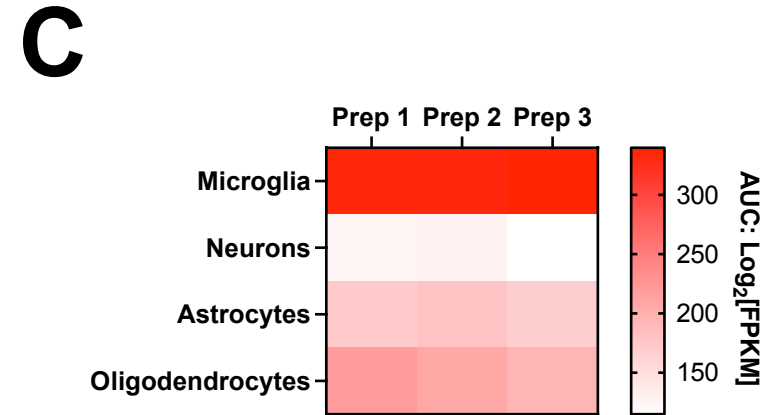

Supplement: Supplementary file 2 — Additional file 2: Figure S2. Depletion of viable microglia from CNS with PLX3397. A IBA-1 immunolabeling indicates complete loss of microglia from cortex of mice fed PLX3397. Shown are representative images from sections of mouse cortex. Scale bar indicates 100 µm and applies to all panels. B Microglia were isolated from cortex of brain from mice fed control chow or PLX3397 and placed into primary culture. Phase contrast images indicate no viable microglia were recovered from mice that were fed PLX3397. Scale bar indicates 100 µm and applies to all panels. C Analysis of gene expression of CD11-isolated cells using gene panels of cell type-specific enriched genes based on gene expression reported in Zhang Y, et al. [52]. Log2 expression data for the top 50 cell type-specific genes for each cell type was summed and plotted as a heatmap. Note the several fold enrichment of microglia-specific genes, indicative of a high-purity of microglia in B. Note that these gene expression data are from mice that were not fed PLX3397, indicating a likely underestimation of the purity of microglia in B. [file 12974_2024_3029_MOESM2_ESM.pdf]

# A

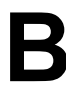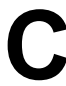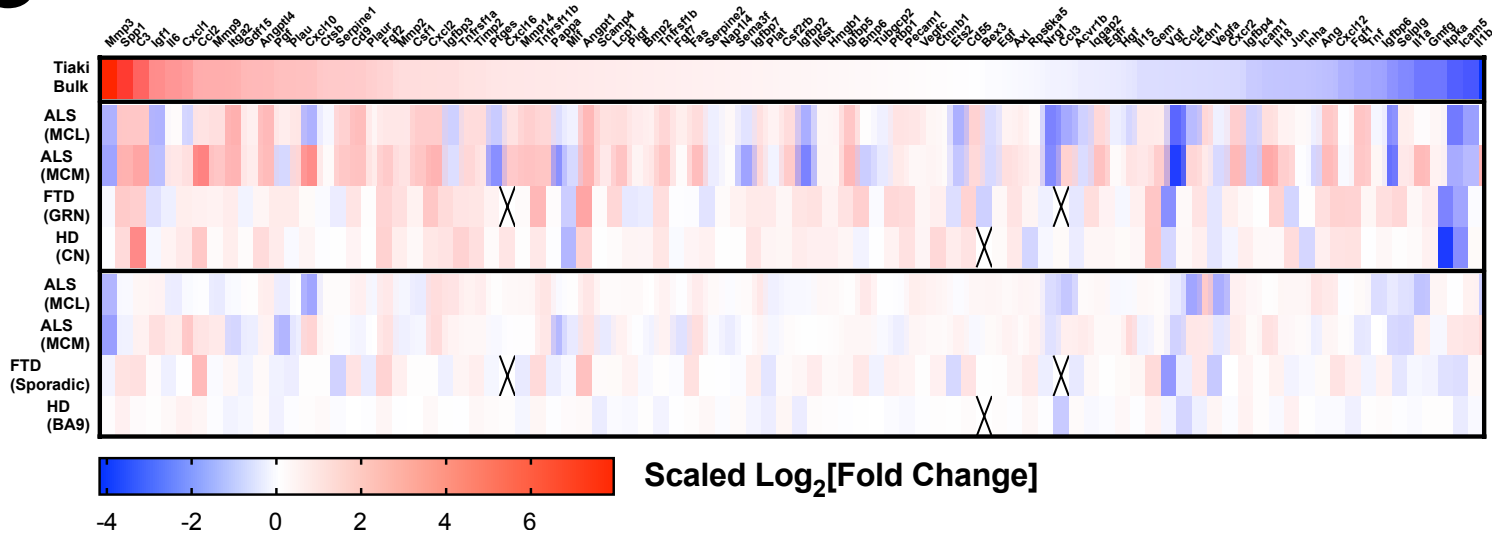

Supplement: Supplementary file 3 — Additional file 3: Figure S3. Brain slice cultures recapitulate human microglial disease signatures. A Heat map of genes from the Human AD microglia signature [24]. Conservation of the HAM gene signature between platform and patient data. Control patient populations show little to no change in these genes. B Heat map of genes from the Lipid-droplet-accumulating microglia signature [25]. Conservation of the LDAM gene signature between platform and patient data. Control patient populations show little to no change in these genes. C Heat map of genes from the human senescence-associated genes (“SenoMayo”) signature [71]. Senescence-associated signature was stronger in the patient data relative to control data. Heat maps indicate Scaled Log2[Fold Change]. ‘X’ indicates a gene that was undetected. Data sources are identical to Fig. 6. [file 12974_2024_3029_MOESM3_ESM.pdf]

# Supplemental Figure 4

A

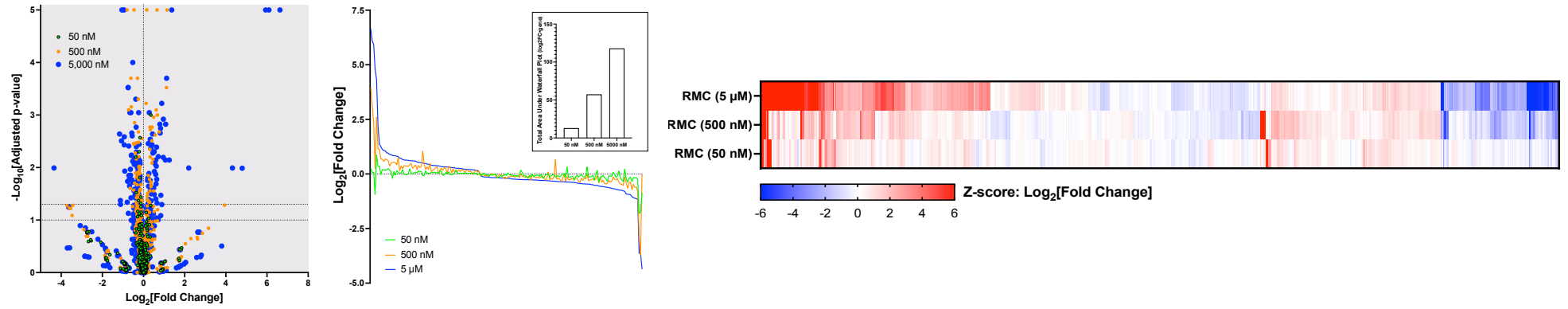

B

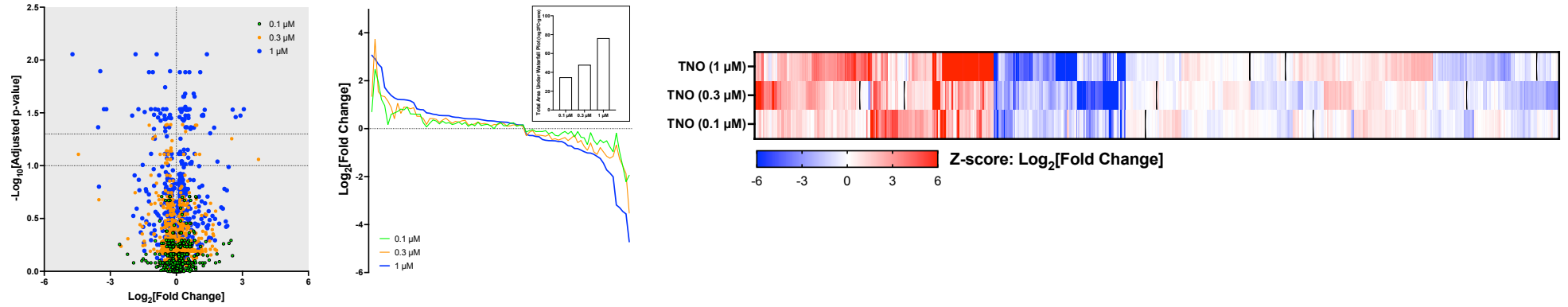

C

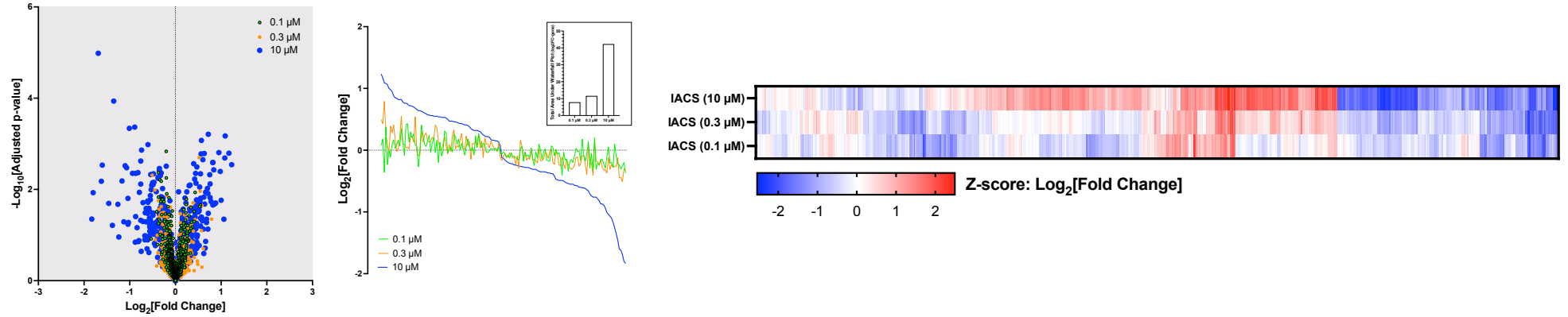

Supplement: Supplementary file 4 — Additional file 4: Figure S4. Dose-dependent modulation of gene expression by RMC-4550, TNO155, and IACS-13909. A RMC-4550 modulated gene expression in a dose-dependent manner. Volcano plot shows increasing number of genes with significant differential expression. Waterfall plot of genes that were significantly differentially expressed at the highest dose of RMC-4550 shows a dose-dependent increase in the area under the curve. Hierarchical clustering of all genes based on Z-scores of Log2-transformed fold changes illustrate dose-responsivity across all genes monitored. B TNO155 modulated gene expression in a dose-dependent manner. Volcano plot shows increasing number of genes with significant differential expression. Waterfall plot of genes that were significantly differentially expressed at the highest dose of TNO155 shows a dose-dependent increase in the area under the curve. Hierarchical clustering of all genes based on Z-scores of Log2-transformed fold changes illustrate dose-responsivity across all genes monitored. C IACS-13909 modulated gene expression in a dose-dependent manner. Volcano plot shows increasing number of genes with significant differential expression. Waterfall plot of genes that were significantly differentially expressed at the highest dose of IACS-13909 shows a dose-dependent increase in the area under the curve. Hierarchical clustering of all genes based on Z-scores of Log2-transformed fold changes illustrate dose-responsivity across all genes monitored. [file 12974_2024_3029_MOESM4_ESM.pdf]
